# Evaluation of the effectiveness of prophylactic sealing of pits and fissures of permanent teeth with fissure sealants - umbrella review

**DOI:** 10.1186/s12903-023-03499-6

**Published:** 2023-10-27

**Authors:** Katarzyna Wnuk, Jakub Świtalski, Wojciech Miazga, Tomasz Tatara, Urszula Religioni, Mariusz Gujski

**Affiliations:** 1 Department of Health Policy Programs, Department of Health Technology Assessment, Agency for Health Technology Assessment and Tariff System, Warsaw, 00032 Poland; 2 School of Public Health, Centre of Postgraduate Medical Education of Warsaw, Kleczewska 61/63, Warsaw, 01826 Poland; 3 Department of Health Economics and Medical Law, Faculty of Health Sciences, Medical University of Warsaw, Warsaw, 01445 Poland; 4 Department of Public Health, Faculty of Health Sciences, Medical University of Warsaw, Warsaw, 02091 Poland

**Keywords:** Dentistry, Permanent dentition, Dental Caries, tooth decay, Pit and fissure sealants, Sealing, Prevention

## Abstract

**Background:**

Tooth decay is an infectious disease which, in its initial phase, leads to the formation of cavities in the teeth through decalcification of the enamel and local tissue destruction. In addition to proper oral hygiene, prophylactic sealing of fissures and cavities with a sealant is a method of preventing the development of caries. The aim of this study is to summarise the effectiveness of fissure sealing of permanent teeth with fissure sealants compared to other preventive methods or no intervention.

**Method:**

An umbrella review was carried out to achieve the purpose of our study. Searches were performed in Medline (via PubMed), Embase (via OVID), and Cochrane Library databases. Quality assessment of the included studies was performed using the AMSTAR2 tool. In addition, a manual search for recommendations/clinical practice guidelines on dental prophylaxis was performed.

**Results:**

204 publications were identified, of which 15 met the inclusion criteria. Based on the results of 3 systematic reviews, there was a statistically significant reduced odds of caries occurrence or development with prophylactic sealing of permanent teeth compared with no intervention – depending on the review and follow-up period odds ratio (OR) ranged from 0.06 [95%CI: (0.01–0.32)] to 0.28 [95%CI: (0.20–0.38)]. In the eight systematic reviews that analysed different sealants, there were no statistically significant differences between the types of materials used for prophylactic tooth sealing. For systematic reviews comparing the efficacy of fissure sealants with fluoride varnish, three reported no statistically significant difference in the efficacy of caries incidence, with only one systematic review based on 1 RCT finding a statistically significant difference in favour of fissure sealants.

**Conclusion:**

Compared to the no intervention, dental sealing is an effective method for the prevention of dental caries. However, it is not possible to conclude conclusively which type of sealant and which of the available prophylactic methods is more effective in preventing caries.

**Supplementary Information:**

The online version contains supplementary material available at 10.1186/s12903-023-03499-6.

## Background

As defined by the International Classification of Diseases 11th Revision (ICD-11: DA08.0), “dental caries is a condition characterised by localised destruction of calcified tissue, initiated on the tooth surface by decalcification of the enamel, followed by the enzymatic lysis of organic structures, resulting in cavity formation” [1].

Caries is an infectious disease, although its pathogenesis is not entirely specific. It is mainly caused by infection with *Streptococcus mutans, Streptococcus sobrinus*, and *Lactobacilli* bacteria. In addition, there is the hypothesis of non-specific plaque, in which caries arises from the metabolic activity of the bacterial biofilm microbiome [2].

Besides below optimal oral hygiene, risk factors for the development of caries include a high frequency of sugar intake and reduced saliva secretion, which may be caused by taking certain medications or due to the presence of other diseases [3].

Based on data from the Institute for Health Metrics and Evaluation/Global Burden of Disease (IHME/GBD), the World Health Organisation (WHO) released the 2022 World Oral Health Report, which identified epidemiologically relevant indicators covering the prevalence of oral diseases – including the incidence of caries in permanent teeth. According to estimates from both organisations, there were more than 2 billion caries cases worldwide in 2019 (N = 2,019,706,083), shaping a global caries prevalence of 28.70%. Considering caries prevalence by region, the highest number of cases were observed in South-East Asia and Western Pacific Regions (nearly 526 million and 464 million cases, respectively) [4]. Furthermore, according to the GBD, the peak incidence of caries is in the 20–24 age group, where it begins to gradually decline in subsequent older age groups, which may be due to teeth loss associated with dental and periodontal diseases [5].

Caries diagnosis begins with a visual assessment of the dried tooth surfaces, with attention being paid to the use of sharply pointed probes, which may disturb the tooth structure during an examination. Next, radiological methods (e.g. digital radiography), optical transillumination (e.g. fibre optic transillumination[FOTI]/digital imaging FOTI [DIFOTI]), fluorescence-based methods (e.g. Diagnodent system), a method based on electrical conductivity or polarisation-sensitive optical coherence tomography (PS-OCT) are used to precisely determine the presence of caries, which are still not visible during physical examination [6].

There are many caries classification systems that can determine the severity, progression, and location of caries. Among the most well-known and widely used are the International Caries Classification and Management System (ICCMS™), the International Caries Detection and Assessment System (ICDAS™), the WHO caries severity classification, and the Black Caries Classification [7].

Areas that favour the development of caries are anatomical pits and fissures in teeth. Consequently, one of the prophylactic methods that can prevent, stop or delay the disease process is the placement of fissure sealants in these tooth structures, resulting in the prevention of food residue accumulation [8]. The materials used in dentistry are fluoride-releasing resin-based sealants (RBS), polyacid-modified resin sealants, glass ionomer cements (GIC), and glass ionomer (GI) sealants [9].

Not only is the prevention, retention, or delay of caries development a measure of the success of fissure sealants, but also the retention rate is an indication of the effectiveness of these materials. It should be noted that, in addition to the structure of the fissure sealant itself, the long-term retention of fissure sealants may depend on a variety of factors, including, for example, adequate maintenance of the dryness of the filling area or the patient’s own cooperation. Accordingly, the retention of fissure sealants may directly influence their effectiveness in preventing carious lesions [10].

Due to the global problem of the high prevalence of dental caries and a large number of systematic reviews on the use of fissure sealants for permanent teeth (in both children and adults), an umbrella review was performed to summarise the evidence of the efficacy of current materials on the market used as prophylactic pit and fissure sealants. Furthermore, considering that the widespread use of fissure sealants could possibly allow a reduction in caries rates and thus prevent early tooth loss and oral disease, this umbrella review analysed the effectiveness of the most common dental fissure sealants in permanent teeth.

Therefore, the aim of the article is to summarise the evaluation of the effectiveness of prophylactic fissure and cavity sealing of permanent teeth using fissure sealants.

## Methods

The search for systematic reviews was based on a detailed protocol developed prior to the work. The protocol was registered on PROSPERO (CRD42023398364) [11].

Systematic reviews meeting the criteria for the following were included in the analysis:

- Population: children and adults receiving prophylactic sealing of permanent teeth.
- Intervention: sealing of teeth with different types of fissure sealants.
- Alternative technologies (comparators): sealing with other sealants, other caries prevention methods, placebo, no intervention.
- Outcome: caries incidence/development/progression, retention, clinical treatment time, patient acceptability.
- Type of included studies: systematic reviews (with or without meta-analysis).

Systematic reviews that analysed combined caries prevention methods, e.g., fissure sealant with fluoride varnish/gel or fissure sealant with resin infiltration, were not included in the umbrella review. Moreover systematic reviews including only resin infiltration was excluded. Our study also did not include publications with significant methodological shortcomings (e.g. lack of correct description of material and method) and inaccuracies in the description of results (e.g. incorrect synthesis of review results, misinterpretation of statistical results.

The following medical information sources were searched: Medline (via PubMed), Embase (via Ovid), The Cochrane Library. The databases were searched on 23 January 2023 according to the search strategies available in the supplementary materials. In addition, manual searches of clinical practice recommendations/guidelines on dental prevention and grey literature were performed (searches included TRIP Database and Google Scholar).

During all stages of the umbrella review, study selection was performed by two analysts working independently (K.W. and J.Ś.). Discrepancies were resolved by consensus, with the involvement of a third independent analyst (W.M.). The most common reasons for excluding studies from analysis were issues related to methodology and results (lack of correct description of material and method, incorrect synthesis of review results, misinterpretation of statistical results) and intervention and population (caries treatment, results relating to deciduous teeth).

The quality and risk of bias assessment of the studies included in the analysis was carried out by reviewing the key domains of the AMSTAR2 systematic review assessment tool [12]. The tool applied identifies publications of the highest quality. To receive the highest rating, a publication must have positive answers to all questions. One shortcoming in a critical domain results in a downgrading of the systematic review to ‘low’. Conversely, two or more failures downgrade the study to ‘critically low’. A quality assessment was performed by two analysts working independently (J.Ś. and W.M.). Discrepancies were resolved by consensus, with the involvement of a third independent analyst (K.W.).

Based on the included publications, data from each publication were summarized for two primary endpoints (caries prophylaxis and fissure seal retention). The studies included in our review were described in terms of characteristics (type and number of studies included, population [age and type of teeth]), interventions used, comparators and endpoints). Information from individual reviews was extracted to Table 1 for caries prevention and Table 2 for retention of fissure sealants, and then collectively descriptive results are presented in the text. For the first endpoint, the analysis disaggregated for comparisons between seals and no intervention, between fissure seals and fluoride, and between types of fissure seals.

Using a spreadsheet, we collected information about the studies included in each of the publications we included in order to investigate whether any reviews covered the same studies. When there was overlap between reviews two authors (K.W. and J.Ś.) discussed cases of overlapping publications. Attention was paid to the described interventions, comparators, search date, type of included studies and risk of bias assessment.

## Results

**Fig. 1:**
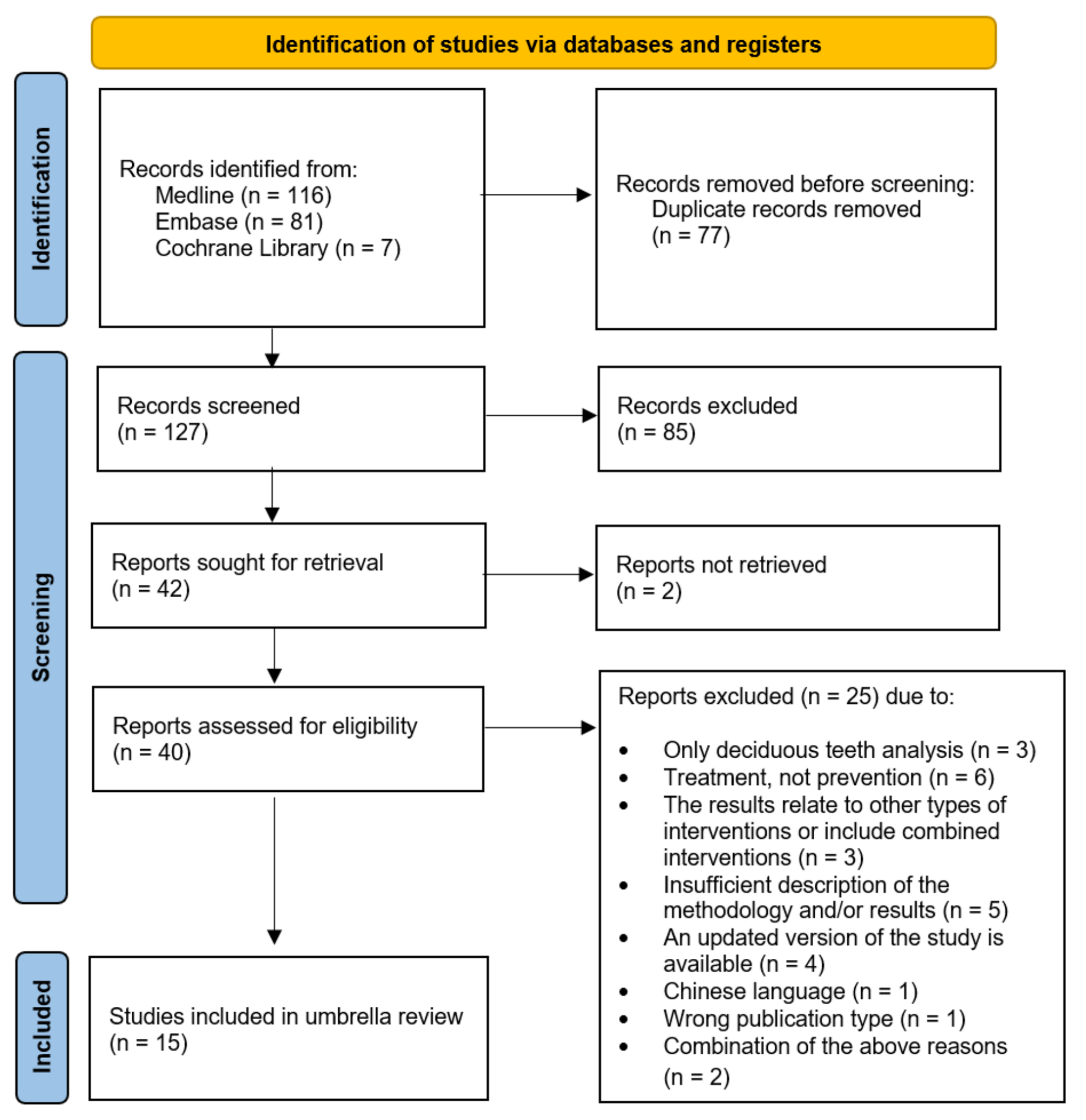
PRISMA flow diagram

The stages of study selection are shown in Fig. 1. A list of included and excluded publications, together with the reasons for exclusion from the review, can be found in the supplementary materials.

The inclusion criteria for the umbrella review were met by the following scientific evidence (n = 15; Bagheri 2022 [13], Alsabek 2021 [14], Kashbour 2020 [15], Kühnisch 2020 [16], Li 2020 [17], Alirezaei 2018 [18], Bagherian 2018 [19], Liang 2018 [20], Ahovuo-Saloranta 2017 [21], Mickenautsch 2016 [22], Wright 2016 [23], Hou 2015 [24], Yengopal 2010 [25], Yengopal 2009 [26], Muller-Bolla 2006 [27]). Details on the methodology of the included systematic reviews can be found in the supplementary material.

The studies found were sufficient to draw conclusions with regard to the efficacy of fissure sealants in caries prevention. In the vast majority of studies, the risk of bias analysis was performed correctly. Carrying out this assessment properly is an important element affecting the quality of the publication and the ability to draw correct conclusions. Moreover, it should be emphasized that the authors of most of the included publications sought to minimize the risk of bias and take into account the risk of bias in results of included studies when interpreting the results of their review (the exception here is the Kühnisch 2020 study [16]). The maximum ratings (overall rating: high) in the AMSTAR2 tool were given to the publications Kashbour 2020 [15], Ahovuo-Saloranta 2017 [21] and Mickenautsch 2016 [22]. Some shortcomings were detected in the remaining publications, most often related to the lack of preparation of the research protocol before starting the study (Bagheri 2022 [13], Kühnish 2020 [16], Li 2020 [17], Alirezaei 2018 [18], Bagherian 2018 [19], Liang 2018 [20], Wright 2016 [23], Hou 2015 [24], Yengopal 2010 [25], Yengopal 2009 [26], Muller-Bolla 2006 [27]) and issues related to the lack of specifying the list of excluded publications together with the reasons for exclusions (Bagheri 2022 [13], Kühnish 2020 [16], Alirezaei 2018 [18], Bagherian 2018 [19], Liang 2018 [20], Wright 2016 [23], Hou 2015 [24]). Another reason for the downgrade was the lack (Bagherian 2018 [19]) or insufficient (Li 2020 [17], Yengopal 2010 [25], Yengopal 2009 [26], Muller-Bolla 2006 [27]) reference to the issue of publication bias. Detailed results of the quality and risk of bias analysis are provided in the supplementary materials.

Of the 195 unique trials reporting primary outcome data and summarized within analyzes relevant to this overview, 56 were included in two or more reviews. The relatively high number of overlapping studies results from the fact that in the publication Kühnisch 2020 [16] a search for scientific evidence was carried out aimed at the retention rate of five groups of sealants. These studies also analyzed other parameters apart from retention rate. These studies also analyzed other parameters apart from retention rate.

### Caries prevention

#### Fissure sealant vs. no sealant

Three meta-analyses (Ahovuo-Saloranta 2017, Wright 2016, Hou 2015) evaluated the impact of the use of fissure sealants in the context of caries prevention [21, 23, 24].

According to the results of the Ahovuo-Saloranta 2017 meta-analysis, the use of resin-based FS (resin-based fissure sealant) in children > 5 years of age reduces the odds of caries occurrence on the occlusal tooth surfaces of permanent molars statistically significantly 2 years after material placement – OR = 0.12 [95%CI: (0.19–0.58); 7 RCTs; N = 1,322; quality of evidence: moderate]. The DMFS (decayed, missing, and filled permanent surfaces) and DFS (decay filled surfaces) rates were also found to be statistically significantly reduced on the basis of 1 RCT, MD (mean difference) = -0.24 [95%CI: (-0.36; -0.12); N = 450] and MD = -0.65 [95%CI: (-0.83; -0.47); N = 276], respectively. In addition, a statistically significant effect of resin-based FS application on the reduced odds of caries was demonstrated at 12, 36, and 48–56 months, with the authors of the publication pointing to a low quality of evidence [21].

The authors of the 2016 Wright meta-analysis indicated that fissure sealing statistically significantly reduced the odds of developing caries over a 2 to 3-year (OR = 0.24 [95%CI: (0.19–0.30)]) and 4 to 7-year follow-up period – OR = 0.21 [95%CI: (0.10–0.44)] [23].

Based on 20 RCTs, the authors of the Hou 2015 meta-analysis showed a statistically significant reduction in the odds of developing caries with fissure sealing of first permanent molars over a follow-up period of 6 months to 5 years, OR = 0.06 [95%CI: (0.01–0.32)] and OR = 0.28 [95%CI: (0.20–0.38)], respectively [24].

#### Fissure sealant vs. fluoride varnish

The studies compared the efficacy of fissure sealants to fluoride varnish in preventing caries incidence [15, 17, 23] and further caries progression [20].

The Kashbour 2020 meta-analysis compared the efficacy of fissure sealants with fluoride varnishes in the prevention of caries in the first permanent teeth. The result of the synthesis of the included studies did not reach statistical significance (OR = 0.67 [95%CI: (0.37–1.19)]). As a result, it is not possible to clearly indicate a more effective of the above-mentioned methods of caries prevention; moreover, the authors of the calculations pointed to the high heterogeneity of the analysed studies, i.e. I^2^ = 84% [15]. Similar conclusions were presented by the authors of Li 2020 analysing 8 RCTs comparing the discussed methods of caries prevention. During a follow-up period of 2–3 years, no statistically significant differences were found in the efficacy of the methods studied in preventing caries cases in first permanent molars (RR = 1.29 [95%CI: (0.95–1.75)]), as well as no statistically significant differences on the occlusal surface of these teeth – RR = 1.33 [95%CI: (0.83–2.11)] [17].

The authors of the 2016 Wright meta-analysis also calculated the effectiveness of fissure sealants in preventing caries over three observation periods of 2–3 years, 4–7 years, and ≥ 7 years. According to the results of this study, the use of fissure sealants at each follow-up period reduces the odds of caries compared to the use of fluoride varnishes OR = 0.27 [95%CI: (0.11–0.69)], OR = 0.19 [95%CI: (0.07–0.51)], and OR = 0.29 [95%CI: (0.17–0.49)], respectively. However, the authors of the meta-analysis themselves pointed out that the result for the longest follow-up period was supported by low-quality evidence [23].

A meta-analysis by Liang 2018, based on secondary caries prevention, showed that the use of resin-based FS on non-cavitied proximal caries, compared to fluoride varnish, statistically significantly reduces the odds of caries progression at 18–24 months (OR = 0.33 [95%CI: (0.19–0.58)]). However, no statistically significant difference was shown between these materials when analysed in subgroups considering caries progression according to caries depth [20].

#### Comparison between fissure sealants

Systematic reviews comparing the efficacy of different types of fissure sealants in preventing caries were also included in the analysis of the publications found in the systematic review. None of the eight identified studies [13, 14, 18, 21–23, 25, 26] analysing this final point indicated a definite statistically significant advantage for any of the fissure sealant types.

In the Mickenautsh 2016 publication, the authors, based on 2 CTs, showed a borderline significant reduced probability of caries incidence when using high-viscosity GI compared to resin-based FS after 60 months of follow-up RR = 0.29 [95%CI: (0.09–0.95)]. In contrast, at shorter follow-up periods (24 to 48 months), there were no statistically significant differences between these materials [22].

The authors of the Yengopal 2010 publication compared RM-GIC with RBS and used the probability of caries absence as an endpoint. On the basis of 1 RCT, a reduced probability of caries absence after 36 months of follow-up by 7% to the detriment of RM-GIC was indicated at the limit of statistical significance – RR = 0.93 [95%CI: (0.88–0.97)] [25]. Similar to the Mickenautsh 2016 publication, the shorter follow-up period (6–24 months) showed no statistically significant difference between the materials analysed.

The characteristics and individual results of studies on caries prevention using fissure sealing methods for permanent teeth are presented below (Table 1).

**Table 1:** Characteristics and results of studies on caries prevention using pit and fissure sealing methods on permanent teeth

Author/year Funding	Number/type of studies included	Population		Intervention		Outcomes	Follow-up (month)	Results (95%CI) [N studies or group; n/N teeth*]
		Description/ type of teeth	Primary sample size (N)	Intervention	Comparator			
*Fissure sealant vs. no sealant*								
Ahovuo-Saloranta 2017 [21] *NIHR via Cochrane Infrastructure*	38 RCT	Children aged 5–16; Occlusal tooth surfaces of permanent molars	7,924	Resin-based FS	No sealants	Caries incidence	12	OR = 0.17 (0.10–0.30) [7 RCT; NS]
							24	OR = 0.12 (0.19–0.58) [7 RCT; N = 1,322]
							36	OR = 0.17 (0.11–0.27) [7 RCT; NS]
							48–56	OR = 0.21 (0.16–0.28) [4 RCT; N = 482]
						DMFS increments	24	MD=-0.24 (-0.36; -0.12) [1 RCT; N = 272 (I); 178 (C)]
						DFS increments	24	MD=-0.65 (-0.83; -0.47) [1 RCT; N = 133 (I); 143 (C)]
				GI	No sealants	DFS increments	24	MD=-0.18 [95%CI: (-0.39-0.03) (1 RCT; N = 261 (I); 143 (C))
Wright 2016 [23] *American Academy of Pediatric Dentistry*	23 RCT	Children and adults; Permanent molars	*Not specified*	FS	No sealants	Caries incidence	2–3 years	OR = 0.24 (0.19–0.30) [9 RCT; n/N = 194/1,799 (I); 584/1,743 (C)]
							4–7 years	OR = 0.21 (0.10–0.44) [3 RCT; n/N = 74/368 (I); 206/384 (C)]
							≥ 7 years	OR = 0.15 (0.08–0.27) [2 RCT; n/N = 62/215 (I); 170/231 (C)]
Hou 2015 [24] *No indicated*	20 RCT (China)	Children and adolescents; First permanent molars	12,187	Resin based FS	No sealants	Caries incidence	6	OR = 0.06 (0.01–0.32) [6 RCT; n/N = 13/2,996 (I); 147/3,026 (C)]
							1 year	OR = 0.10 (0.05–0.21) [17 RCT; n/N = 277/8,142 (I); 796/8,017 (C)]
							2 year	OR = 0.16 (0.09–0.26) [15 RCT; n/N = 257/6,868 (I); 873/6,729 (C)]
							3 year	OR = 0.21 (0.13–0.32) [13 RCT; n/N = 321/6,086 (I); 937/5,971 (C)]
							4 year	OR = 0.18 (0.05–0.62) [3 RCT; n/N = 217/1,837 (I); 560/1,676 (C)]
							5 year	OR = 0.28 (0.20–0.38) [2 RCT; n/N = 63/843 (I); 189/843 (C)]
*Fissure sealant vs. fluoride varnish*								
Wright 2016 [23] *American Academy of Pediatric Dentistry*	23 RCT	Children and adults; Permanent molars	*Not specified*	FS	Fluoride varnishes	Caries incidence	2–3 years	OR = 0.27 (0.11–0.69) [3 RCT; n/N = 66/855 (I); 364/860 (C)]
							4–7 years	OR = 0.19 (0.07–0.51) [2 RCT; n/N = 46/228 (I); 131/244 (C)]
							≥ 7 years	OR = 0.29 (0.17–0.49) [1 RCT; n/N = 30/113 (I); 113/129 (C)]
Kashbour 2020 [15] *NIHR via Cochrane Infrastructure*	11 RCT	Children aged 5–10; Occlusal tooth surfaces of permanent first molars	3,374	Resin-based FS	Fluoride varnish	Caries incidence	2–3 year	OR = 0.67 (0.37–1.19) [4 RCT: 1 split-mouth paired data and 3 parallel-group; N = 1,683]
Li 2020 [17] *National Natural Science Foundation of China*	8 RCT	Children aged 6–9; First permanent molars	3,289 (6,878 FPMs)	FS (resin-based or glass-ionomer)	Fluoride varnish (22,600 ppm)	Caries incidence	2–3 year	*Children* RR = 1.12 (0.60–2.09) [2 RCT; n/N = 88/533 (I); 91/539 (C)]
								*FPMs* RR = 1.29 (0.95–1.75) [6 RCT; n/N = 405/3,452 (I); 339/3,426 (C)]
								*FPMs’occlusal surfaces* RR = 1.33 (0.83–2.11) [4 RCT; n/N = 299/3,279 (I); 256/3,272 (C)]
						DMFS		*Occlusal FPMs* MD = 0.13 (-0.09; 0.34) [4 RCT; N = 905 (I); 882 (C)]
Liang 2018 [20] *Science and Technology Program of Shenzhen and Guangzhou*	8 RCT *Split-mouth design*	Children, adolescents, adults; Non-cavitated proximal caries	534 (I) 490 (C)	Resin-based FS (micro-invasive interventions)	Fluoride varnish	Caries progression	18–24	OR = 0.33 (0.19–0.58) [3 RCT; n/N = 34/147 (I); 64/142 (C)]
						Caries progression (depth)	24 overall	OR = 0.50 (0.19–1.28) [3 RCT; n/N = 9/75 (I); 15/70 (C)]
							24	Enamel OR = 0.62 (0.13–3.00) [1 RCT; n/N = 3/38 (I); 4/33 (C)]
							18	Enamel-dentin junction OR = 0.44 (0.09–2.15) [1 RCT; n/N = 4/13 (I); 7/14 (C)]
							18	Dentin OR = 0.43 (0.07–2.63) [1 RCT; n/N = 2/24 (I); 4/23 (C)]
				GIC (micro-invasive interventions)	Fluoride gel	Caries progression	18–24	OR = 0.13 (0.01–2.65) [1 RCT; n/N = 0/41 (I); 3/41 (C)]
*Comparison between fissure sealants*								
Bagheri 2022 [13] *Not indicated*	8 RCT	Children; First molars	1,355 (I) 1,390 (C)	Filled resin-based FS	Unfilled resin-based FS	Caries development	6	OR = 2.48 (0.567–10.843) (5 RCT/6 group; NS]
							12	OR = 0.995 (0.441–2.224) [8 RCT/12 groups; NS]
							> 12	OR = 2.764 (0.825–9.262) [3 RCT/groups; NS]
Alsabek 2021 [14] *No funding*	13 RCT	Permanent	1,487	Hydrophilic resin-based sealant	Other fissure sealants	Caries incidence	6	RR = 0.97 (0.91–1.03) [4 RCT; 174/196 (I); 180/196 (C)]
							12	RR = 0.97 (0.91–1.03) [5 RCT; 250/294 (I); 258/294 (C)]
Alirezaei 2018 [18] *Not indicated*	31 RCT	Permanent molars	7,168 (I) 7,536 (C)	Resin-based FS	GIC	Caries development	6	OR = 0.938 (0.647–1.359) [20 RCT/22 groups; NS]
					High-viscosity GIC			OR = 0.852 (0.586–1.238) [6 RCT/8 groups; NS]
					Low-viscosity GIC			OR = 0.773 (0.469–1.274) [10 RCT/groups; NS]
Ahovuo-Saloranta 2017 [21] *NIHR via Cochrane Infrastructure*	38 RCT	Children aged 5–16; Occlusal tooth surfaces of permanent molars	7,924	Low-viscosity and resin modified GI	Resin-based FS	Caries incidence	12	OR = 1.47 (0.64−3.37) [6 RCT; NS]
				Low-viscosity GI		Caries incidence	24	OR = 1.67 (0.87–3.20) [10 RCT; NS]
				High-viscosity GI		Caries incidence	24	OR = 1.36 (0.56–3.22) [2 RCT; NS]
				Resin modified GI		Caries incidence	24	OR = 2.92 (1.77–4.81) [2 RCT; NS]
				GI	Resin-based FS	DFS increments	24	MD = 0.47 (0.31; 0.63) [1 RCT; N = 261 (I); 133 (C)]
Mickenautsch 2016 [22] *No funding*	7 CT (11 datasets)	Children aged 5–11 (mean 7.8); First permanent molars	*Not specified*	High-viscosity GI	Resin-based FS	Caries incidence	24	RR = 1.36 (0.66–2.78) [4 CT; n/N = 24/808 (I); 24/934 (C)]
							36	RR = 0.90 (0.49–1.67) [3 CT; n/N = 15/261 (I); 27/339 (C)]
							48	RR = 0.62 (0.31–1.21) [2 CT; n/N = 13/488 (I); 22/519 (C)]
							60	RR = 0.29 (0.09–0.95) [2 CT; n/N = 3/101 (I); 13/104 (C)]
Wright 2016 [23] *American Academy of Pediatric Dentistry*	23 RCT	Children and adults; Permanent molars	*Not specified*	GI sealant	Resin-based FS	Caries incidence	2–3 years	OR = 0.71 (0.32–1.57) [10 RCT; n/N = 179/2,727 (I); 141/2,014 (C)]
							4–7 years	OR = 0.37 (0.14-1.00) [2 RCT; n/N = 6/61 (I); 37/84 (C)]
				GI sealant	Resin-modified GI		2–3 years	OR = 1.41 (0.65–3.07) [1 RCT; n/N = 27/172 (I); 20/172 (C)]
				Resin-modified GI sealant	Polyacid-modified resin sealants		2–3 years	OR = 0.44 (0.11–1.82) [1 RCT; n/N = 3/97 (I); 6/89 (C)]
				Polyacid-modified resin sealants	Resin-based sealants		2–3 years	OR = 1.01 (0.48–2.14) [2 RCT; n/N = 16/159 (I); 16/163 (C)]
Yengopal 2010 [25] *No indicated*	6 RCT (19 datasets)	Children aged 5–11; Permanent molars	491 teeth; 227 (I) 264 (C)	Resin-modified GIC	Resin-based sealants	Caries absence	6	RR = 0.98 (0.95-1.00) [3 RCT; N = 227 (I); 264 (C)]
							12	RR = 1.00 (0.96–1.04) [4 RCT; N = 341 (I); 378 (C)]
							24	RR = 1.01 (0.84–1.21) [2 RCT; N = 227 (I); 264 (C)]
							36	RR = 0.93 (0.88–0.97) (1 RCT; N = 136 (I); 136 (C)]
Yengopal 2009 [26] *No indicated*	8 RCT, 3 SR	Children aged 6–11; Permanent molars	827 (I) 822 (C)	GIC	Resin-based FS	Caries absence	1–3 years	OR = 0.96 (0.62–1.49) [6 RCT; n/N = 784/827 (I); 781/822 (C)]

* n = case; N = number of teeth in the intervention or control group; (I) – group examined; (C) – group control

CI – confidence interval; CT – clinical trial; DFS – decay filled surfaces, DMFS – decay, missing and filled permanent surfaces; FPMs – first permanent molars; FS – fissure sealant, GIC – glass ionomer cement; GI – glass-ionomer; NS – not specified; OR – odds ratio; ppm – parts per million; RCT – randomized controlled trial; RR – relative risk; SR systematic review

### Retention of fissure sealants

Seven publications [13, 14, 16, 18, 19, 23, 27] that described the retention of different types of fissure sealants were included in the analysis, with endpoints that varied from study to study.

#### Fissure sealant vs. no sealant

In the Kühnisch 2020 meta-analysis, the authors compared pooled retention rate estimates (RREs) of five groups of sealants, i.e. primed, auto-polymerising, light-polymerising, fluoride-releasing and glass-ionomer sealant. According to the results of this publication, the shortest retention is characterised by primed and glass-ionomer sealants with RRE = 14.1% [95%CI: (5.7–22.7)] and RRE = 43.2% [95%CI: (30.5–55.8)] at a 2-year follow-up and RRE = 8.4% [95%CI: (10.2–15.8) and RRE = 33.10% [95%CI: (20.8–45.4)] at a 3-year follow-up, respectively. The longest retention was demonstrated by auto-polymerising and light-polymerising sealants showing RRE = 70.0% [95%CI: (48.0–92.1)] and RRE = 57.8% [95%CI: (38.6–76.9)], respectively, over a 5-year follow-up period [16].

#### Comparison between conventional materials

In contrast, the authors of the Bagherian 2018 publication compared the retention rate for conventional flowable composite versus conventional sealants. According to a meta-analysis based on nine RCTs, a statistically significantly higher retention rate was shown for flowable composite compared to conventional sealants – OR = 2.387 [95%CI: (1.047–5.444)] [19].

#### Comparison between resin-based sealants

The authors of the publications Bagheri 2022 [13] and Alsabek 2021 [14] indicated that there were no statistically significant differences in retention rates between filled and unfilled sealants, respectively, and between hRBS and other fissure sealants such as conventional resin or glassimonomer-based fillings.

The Muller-Bolla 2006 meta-analysis compared the probability of maintaining complete retention when using different subtypes of resin-based sealants (light-cured RBS, auto-polymerised RBS, fluoride-containing RBS). According to the results of the publication, there was no statistically significant difference in the maintenance of complete retention for light-cured RBS compared to auto-polymerised RBS between 6 and 60 months. However, a statistically significant 20% reduced probability of maintaining complete retention was found for fluoride-containing RBS compared to light-cured RBS at 48 (2 RCTs) and 54 (1 RCT) months, respectively – RR = 0.80 [95%CI: (0.72–0.89)] and RR = 0.80 [95%CI: (0.68–0.93)] [27].

#### Comparison beetween resin-based fissure sealants and glass-ionomer sealants

In the Alirezaei 2018 publication, the authors compared the retention rate for resin-based FS versus GIC (including high-viscosity, low-viscosity, and resin-modified GIC) at 6 months after application. Based on 28 RCTs, there was a statistically significantly higher retention rate for resin-based FS compared to GIC – OR = 6.006 [95%CI: (3.226–11.183)]. Furthermore, when analysed in subgroups against GIC subtypes, higher retention rates were also obtained for resin-based FS compared with high-viscosity GIC (OR = 4.091 [95%CI: (1.680–9.963)]), low-viscosity GIC (OR = 5.093 [95%CI: (2.390–10.852)]) and resin-modified GIC (OR = 16.785 [95%CI: (2.355–119.632)]) [18].

In the Wright 2016 meta-analysis, the endpoint for maintenance of the different types of fissure sealants was a lack of retention. In line with the publication’s results, statistical significance was obtained with a calculation indicating a higher odds of loss of retention with GI sealant compared to resin-based FS over a 2-3-year follow-up period (OR = 5.06 [95%CI: (1.81–14.13)]) and a result indicating a higher odds of loss of retention with GI sealant compared to resin-modified GI over a 2-3-year follow-up period OR = 3.21 [95%CI: (1.87–5.51)] [23].

The characteristics and individual results of the study regarding the retention of specific types of permanent tooth fissure sealants are presented below (Table 2).

**Table 2:** **Results of studies on the retention of different types of pit and fissure sealers on permanent teeth**

Author/year Funding	Number/type of studies included	Population		Intervention		Outcomes	Follow-up	Results (95%CI) [N studies or group; n/N teeth*]
		Description/ type of teeth	Primary sample size (N)	Intervention	Comparator			
*Fissure sealants vs. no sealant*								
Kühnisch 2020 [16] *Not indicated*	75 CT	Children and adolescents aged < 21; Permanent molars	*Not specified*	Prime sealants	*Not applicable*	Retention rate estimate %	2 year	RRE = 43.2 (30.5–55.8) [11 CT; N = 605]
							3 year	RRE = 33.10 (20.8–45.4) [4 CT; N = 316]
				Auto-polymerizing sealants			2 year	RRE = 80.8 (72.2–89.5) [22 CT; N = 4,192]
							3 year	RRE = 73.4 (67.5–79.3) [15 CT; N = 3,270]
							5 year	RRE = 70.0 (48.0–92.1) [3 CT; N = 486]
				Light-polymerizing sealants			2 year	RRE = 69.4 (60.0–76.7) [24 CT; N = 2,615]
							3 year	RRE = 83.1 (75.6–90.7) [10 CT; N = 1,860]
							5 year	RRE = 57.8 (38.6–76.9) [4 CT; N = 528]
				Fluoride-releasing sealants			2 year	RRE = 63.8 (53.1–74.4) [15 CT; N = 1,570]
							3 year	RRE = 86.4 (73.4–99.3) [3 CT; N = 460]
							5 year	RRE = 43.3 (16.2–70.3) [2 CT; N = 333]
				Glass-ionomer sealants			2 year	RRE = 14.1 (5.7–22.7) [23 CT; N = 3,292]
							3 year	RRE = 8.4 (10.2–15.8) [12 CT; N = 2,136]
							5 year	RRE = 1.6 (0.0–28.1) [2 CT; N = 256]
*Comparison between conventional materials*								
Bagherian 2018 [19] *Not indicated*	11 RCT	Permanent molars	599 (I) 597 (C)	Conventional flowable composite	Conventional sealants	Retention rate	1–2 years	OR = 2.387 (1.047–5.444) [9 RCT; N = 599 (I); 597 (C)]
*Comparison between resin-based sealants*								
Bagheri 2022 [13] *Not indicated*	8 RCT	Children; First molars	1,355 (I) 1,390 (C)	Filled resin-based FS	Unfilled resin-based FS	Retention rate	6 month	OR = 1.010 (0.704–1.447) [14 RCT/17 groups; NS]
							12 month	OR = 1.042 (0.700–1.551) [18 RCT/25 groups; NS]
							> 12 month	OR = 1.429 (0.695–2.939) [6 RCT/17 groups; NS]
Alsabek 2021 [14] *No funding*	13 RCT	Permanent	1,487	Hydrophilic resin-based sealant	Other fissure sealants	Retention rate	6 month	RR = 1.04 (0.97–1.11) [5 RCT; n/N = 212/236 (I); 204/236 (C)]
							12 month	RR = 1.03 (0.89–1.19) [5 RCT; n/N = 246/294 (I); 243/294 (C)]
Muller-Bolla 2006 [27] *Not indicated*	16 RCT	Children aged 5–10 Permanent molars	4,944 teeth	Light-cured resin based sealants	Auto-polymerized resin-based sealants	Complete retention	6 month	RR = 0.98 (0.87–1.11) [2 RCT; n/N = 132/181 (I); 128/172 (C)]
							12 month	RR = 0.95 (0.91–1.00) [6 RCT; n/N = 641/815 (I); 585/715 (C)]
							24 month	RR = 0.99 (0.93–1.06) [4 RCT; n/N = 426/553 (I); 375/485 (C)]
							36 month	RR = 0.99 (0.92–1.07) [2 RCT; n/N = 324/432 (I); 323/428 (C)]
							60 month	RR = 0.79 (0.60–1.04) [1 RCT; n/N = 43/90 (I); 49/81 (C)]
				Fluoride-containing resin-based sealants	Light-cured resin based sealants		12 month	RR = 1.01 (0.96–1.06) [5 RCT; n/N = 380/449 (I); 395/468 (C)]
							24 month	RR = 0.95 (0.79–1.15) [1 RCT; n/N = 20/23 (I); 31/34 (C)]
							48 month	RR = 0.80 (0.72–0.89) [2 RCT; n/N = 177/270 (I); 229/282 (C)]
							54 month	RR = 0.80 (0.68–0.93) [1 RCT; n/N = 90/144 (I); 94/120 (C)
*Comparison beetween resin-based fissure sealants and glass-ionomer sealants*								
Alirezaei 2018 [18] *Not indicated*	31 RCT	Permanent molars	7,168 (I) 7,536 (C)	Resin-based FS	GIC	Retention rate	6 month	OR = 6.006 (3.226–11.183) [28 RCT/32 groups; NS]
					High-viscosity GIC			OR = 4.091 (1.680–9.963) [4 RCT; NS]
					Low-viscosity GIC			OR = 5.093 (2.390–10.852) [18 RCT/21 groups; NS]
					Resin-modified GIC			OR = 16.785 (2.355–119.632) [5 RCT/groups; NS]
Wright 2016 [23] *American Academy of Pediatric Dentistry*	23 RCT	Children and adults; Permanent molars	*Not specified*	GI sealant	Resin-based FS	Lack of retention	2–3 years	OR = 5.06 (1.81–14.13) [10 RCT; n/N = 1,875/2,727 (I); 596/2,014 (C)]
							4–7 years	OR = 2.08 (0.15–27.95) [2 RCT; n/N = 46/61 (I); 50/84 (C)]
				GI sealant	Resin-modified GI		2–3 years	OR = 3.21 (1.87–5.51) [1 RCT; n/N = 149/172 (I); 115/172 (C)]
				Resin-modified GI sealant	Polyacid-modified resin sealants		2–3 years	OR = 1.17 (0.52–2.66) [1 RCT; n/N = 15/97 (I); 12/89 (C)]
				Polyacid-modified resin sealants	Resin-based sealants		2–3 years	OR = 0.87 (0.12–6.21) [2 RCT; n/N = 15/159 (I); 15/163 (C)]

* n = case; N = number of teeth in the intervention or control group; (I) – group examined; (C) – group control

CI – confidence interval; CT – clinical trials; FS – fissure sealant, GI – glass-ionomer; GIC – glass ionomer cement; NS – not specified; OR – odds ratio; RCT – randomized controlled trial; RRE – retention rate estimate; RR – relative risk;

## Discussion

Based on the results of studies found in the systematic review, the effectiveness of fissure sealants in preventing the occurrence or development of caries was assessed. Publications comparing, among other things, sealants with no intervention and other preventive methods were analysed, as well as studies comparing different types of fissure sealants.

The results of the studies clearly indicate that, compared to no intervention, sealing permanent teeth reduces the odds of caries occurrence or development [21, 23, 24]. When analysing comparisons of different types of sealants, it should be noted that the vast majority of studies did not report statistically significant differences [13, 14, 18, 21, 23, 26]. The exception is the Mickenautsh 2016 study, which compared a high-viscosity glass-ionomer with a resin-based fissure sealant. Although no differences in caries incidence were observed at 24-, 36- and 48-month follow-up, a borderline significant difference in favour of high-viscosity glass-ionomer emerged after 60 months [22]. In contrast, in the Yengopal 2010 study, which compared resin-modified glass-ionomer cement with resin-based sealants, a statistically significant difference in favour of the former sealant was observed after 36 months of follow-up. However, it should be noted that the result came from only 1 RCT and was on the borderline of statistical significance. At 6-, 12- and 24-month follow-up, no differences in favour of one or the other sealant were noted [25].

In this regard, it should be emphasised that there are significant differences when comparing RBS with GIC in terms of retention rate. The results of the Alirezaei 2018 meta-analysis show a clearly superior retention rate in favour of RBS. This may be influenced by, among other things, “higher wear resistance and compressive strength of RBSs, as well as their micromechanical bonding to the tooth structure after etching procedures” [18]. On the other hand, the 2016 Wright meta-analysis, which analysed the lack of retention rate, also showed an advantage in favour of RBSs (with GI sealant the risk of loss of retention was higher) [23].

Meanwhile, research findings suggest that the caries prevention effect of GIC-based sealant may not be directly related to retention. A 2013 study by Mickenautsch found that the risk of loss of complete retention material increased the risk of caries development with RBS, but not with GIC-based sealant [18, 28].

According to the results of studies comparing fissure sealant with fluoride varnish, it is not possible to determine with certainty which of these methods is more effective. Three studies reported no statistically significant differences between fissure sealant and fluoride varnish in terms of caries incidence [15, 17] or caries progression [20]. In contrast, one study reported a significant difference in favour of fissure sealant in all observation periods analysed [23].

There were no systematic reviews in this umbrella review that met the inclusion criteria in terms of clinical treatment time and patient acceptability (as outcomes).

To complete the analysis, databases and websites of scientific societies were manually searched for clinical practice guidelines on tooth sealing. Publications from the last 10 years were searched for. The conclusions of the recommendations are presented below.

Found documents clearly indicate that sealants can be used on permanent teeth as a caries prevention method [8, 29–34]. The 2016 guidelines of the American Academy of Pediatric Dentistry and American Dental Association indicate that the use of sealants is recommended for occlusal surfaces and non-cavitated occlusal carious lesions in children and adolescents. It was also emphasised that it is not possible to give a clear indication of the type of sealant that should be used [8].

The European Academy of Paediatric Dentistry guidelines emphasise that “pit and fissure sealing prevents new occlusal caries in permanent molars and is able to arrest existing non-cavitated lesions” [31].

The 2014 Scottish Intercollegiate Guidelines Network indicated that ‘resin-based fissure sealants should be applied to the permanent molars of all children as early after eruption as possible’ and ‘glass-ionomer sealants may be considered if the application of a resin-based sealant is not possible’ [33]. The document and the recommendation in the section on tooth sealing were based on the Cochrane review Ahovuo-Saloranta 2013 [35]. It concluded that it was not possible to draw any conclusions about the superiority of resin-based fissure sealants over glass ionomer sealants. Fifteen studies comparing the above-mentioned types of sealants were analysed, in which 4 found better caries reductions for resin-based sealants, 2 found better caries reductions for glass ionomers, and 9 reported no differences. However, some variations in retention were noted that may indicate a benefit for resin sealants at a 36–48 month follow-up. It was emphasised, though, that this is not a basis for drawing a simple conclusion regarding the superiority of this type of sealant. An update of the above-mentioned publication [21] was included in our systematic review. The main conclusions of the study remain unchanged from the 2013 version.

Considering the above data, it is worth noting the very small number of available guidelines addressing tooth sealing issues with a global reach (issued by major organisations/scientific societies in English, with easy access to the full text of the publication) published in the last 10 years. The most recent guidelines comprehensively addressing the topic of tooth sealing were published in 2016. It would therefore seem reasonable to issue an update of the guidelines on the sealing of permanent teeth.

The economic factor should also be taken into account when analysing issues related to dental prevention. An Akinlotan 2017 systematic review targeted at the cost-effectiveness analysis of sealants compared to no intervention or other caries prevention was found [36]. It identified 2 studies analysing the cost-effectiveness of sealants compared with no intervention. Based on a study that analysed incremental cost per DMFS averted over 5 years, the result was estimated to be $41.96 [36, 37]. Another study presented incremental cost to transition from restored tooth (utility = 0.81) to sealed sound tooth (utility = 1) over 4 years, and the result was estimated to be $45.19-$102.81 per 0.19 QATY (quality-adjusted tooth years) [36, 38].

In studies comparing dental sealing with other methods of caries prevention, it was not possible to conclude definitively which method was more cost-effective (some studies indicated that sealing was more cost-effective and others that it was less so). Only one study found was comparing the use of resin to glass-ionomer sealants, which indicated that light-emitting diode thermocured glass-ionomer sealants were more costly but also more effective than the composite resin group [36, 39]. However, it should be considered that the cost-effectiveness of sealants may depend on the conditions of delivery, as also pointed out by the authors of the systematic review Akinlotan 2017.

When analyzing other issues related to the effectiveness of sealing of pits and fissures, it should also be borne in mind that elements such as using adhesive systems and preparation of tooth surfaces prior to fissure sealants placement may be important. In a systematic review, one of the assumptions of which was “evaluate fissure sealant retention with and without the use of an adhesive system”, it was shown that the use of adhesive systems beneath fissure sealants can significantly increase retention [40]. Results from systematic reviews of preparation of tooth surfaces prior to fissure sealants placement also indicate potential retention benefits. In one of the reviews, the authors indicated that the preparation method (e.g. air abrasion, carbon dioxide laser, round bur on slow-speed handpiece) before acid etching had a significant positive effect on fissure sealant retention. However, the authors emphasized that preparation alone cannot be a complete substitute for conventional acid etching before sealant placement. The two main reasons were higher costs of these techniques and the increased predisposition to cars because of the opening of fissures after sealant loss. At the same time, it was indicated that no significant differences were found between the preparation-only method and the conventional acid-etching method in terms of fissure sealant retention [41]. In another study, it was indicated that laser preparation was a safe, effective and highly-acceptable method of enamel preparation before sealant placement, and the retention rate after laser preparation alone was comparable to that of acid-etching preparation [42]. However, it should be borne in mind that the number of high-quality studies in the discussed areas is small, which is a serious limitation of the possibility of unequivocal conclusions about the effect of the methods described in this paragraph.

## Conclusions

Dental sealing is an effective method for the prevention of dental caries. Compared to no intervention, it reduces the risk of caries occurrence/development. On the basis of the systematic reviews found, it is not possible to conclude clearly which type of sealant is most effective in preventing caries. There are data indicating better results in terms of the retention rate of resin-based FS, but this cannot be the basis for concluding an absolute superiority of this type of material. It is also not possible to state conclusively whether dental sealing is more effective than other methods of caries prevention (e.g. fluoride varnish).

Further studies (optimally RCTs) comparing retention over a long follow-up period (more than 5 years) are required, in particular regarding the comparison of resin-based FS retention with GI sealant and the effect of retention on caries risk for individual sealants.

### Review Limitations

Only publications in English were included in the review. The studies found were characterised by high heterogeneity and used varied methods of presenting the analysed data. The results of the systematic reviews are limited due to the small number of high-quality studies (inference regarding the analysed endpoint was often based on the results of a single RCT). It should also be considered that the populations of children, adolescents and adults were not analysed separately (the results of permanent teeth sealing were presented, with no breakdown by the age at which the service was provided).

### Electronic supplementary material

Below is the link to the electronic supplementary material.

Supplementary Material 1

## Data Availability

All data are available from the corresponding author.

## List of abbreviations

CT: clinical trials
DOFOTI: digital imaging FOTI
FOTI: fibre optic transillumination
GI: glass ionomer sealants
GIC: glass ionomer cements
hRBS: hydrophilic resin-based fissure sealants
ICCMS: International Caries Classification and Management System
ICDAS: International Caries Detection and Assessment System
IHME/GBD: Institute for Health Metrics and Evaluation/Global Burden of Disease
PC: OCT-polarisation-sensitive optical coherence tomography
RBS: releasing resin-based sealants
RCT: Randomised Controlled Trial
WHO: World Health Organisation
