# Supplementary material for: Evaluation of the effectiveness of prophylactic sealing of pits and fissures of permanent teeth with fissure sealants - umbrella review
Source: BMC Oral Health. 2023 Oct 27;23:806. doi: 10.1186/s12903-023-03499-6 (PMC10612169; doi:10.1186/s12903-023-03499-6)
Supplement: Supplementary file 1 — Supplementary Material 1 [file 12903_2023_3499_MOESM1_ESM.docx]

**Search strategy Medline (via PubMed)**

| **ID** | **Keyword** | **Result** |
| --- | --- | --- |
| #1 | "Pit and Fissure Sealants"[Mesh] | 3556 |
| #2 | "Fissure seal*"[Title/Abstract] | 1918 |
| #3 | "Dental sealant*"[Title/Abstract] | 447 |
| #4 | "Compomer sealant*"[Title/Abstract] | 4 |
| #5 | "Composite sealant*"[Title/Abstract] | 30 |
| #6 | "Glass Ionomer Cements"[Mesh] | 7271 |
| #7 | "Glass ionomer*"[Title/Abstract] OR glassionomer*[Title/Abstract] | 7136 |
| #8 | ("Glass Ionomer Cements"[Mesh]) OR ("Glass ionomer*"[Title/Abstract] OR Glassionomer*[Title/Abstract]) | 9427 |
| #9 | Sealant*[Title/Abstract] | 7576 |
| #10 | (("Glass Ionomer Cements"[Mesh]) OR ("Glass ionomer*"[Title/Abstract] OR Glassionomer*[Title/Abstract])) AND (Sealant*[Title/Abstract]) | 462 |
| #11 | ((((("Pit and Fissure Sealants"[Mesh]) OR ("Fissure seal*"[Title/Abstract])) OR ("Dental sealant*"[Title/Abstract])) OR ("Compomer sealant*"[Title/Abstract])) OR ("Composite sealant*"[Title/Abstract])) OR ((("Glass Ionomer Cements"[Mesh]) OR ("Glass ionomer*"[Title/Abstract] OR Glassionomer*[Title/Abstract])) AND (Sealant*[Title/Abstract])) | 4212 |
| #12 | ((((("Pit and Fissure Sealants"[Mesh]) OR ("Fissure seal*"[Title/Abstract])) OR ("Dental sealant*"[Title/Abstract])) OR ("Compomer sealant*"[Title/Abstract])) OR ("Composite sealant*"[Title/Abstract])) OR ((("Glass Ionomer Cements"[Mesh]) OR ("Glass ionomer*"[Title/Abstract] OR Glassionomer*[Title/Abstract])) AND (Sealant*[Title/Abstract])) Filters: Meta-Analysis, Systematic Review | 116 |

**Search strategy Cochrane Library**

| **ID** | **Keyword** | **Result** |
| --- | --- | --- |
| #1 | MeSH descriptor: [Pit and Fissure Sealants] explode all trees | 414 |
| #2 | ("Fissure seal*"):ti,ab,kw | 71 |
| #3 | ("Dental sealant*"):ti,ab,kw | 55 |
| #4 | ("Compomer sealant*"):ti,ab,kw | 1 |
| #5 | ("Composite sealant*"):ti,ab,kw | 12 |
| #6 | MeSH descriptor: [Glass Ionomer Cements] explode all trees | 790 |
| #7 | ("Glass ionomer*" OR glassionomer*):ti,ab,kw | 1655 |
| #8 | #6 OR #7 | 1662 |
| #9 | (Sealant*):ti,ab,kw | 1906 |
| #10 | #8 AND #9 | 236 |
| #11 | #1 OR #2 OR #3 OR #4 OR #5 OR #10 | 610 |
| #12 | #1 OR #2 OR #3 OR #4 OR #5 OR #10, in Chochrane Reviews | 7 |

**Search strategy Embase (via Ovid)**

| **ID** | **Keyword** | **Result** |
| --- | --- | --- |
| 1 | exp fissure sealant/ | 2674 |
| 2 | (Fissure adj6 seal*).ab,kw,ti. | 1438 |
| 3 | (Dental adj3 sealant*).ab,kw,ti. | 554 |
| 4 | (Compomer adj4 sealant*).ab,kw,ti. | 14 |
| 5 | (Composite adj4 sealant*).ab,kw,ti. | 151 |
| 6 | exp glass ionomer/ | 7632 |
| 7 | (Glass ionomer* or glassionomer*).ab,kw,ti. | 6452 |
| 8 | 6 or 7 | 8707 |
| 9 | "Sealant*".ab,kw,ti. | 7893 |
| 10 | 8 and 9 | 436 |
| 11 | 1 or 2 or 3 or 4 or 5 or 10 | 3294 |
| 12 | limit 11 to (meta analysis or "systematic review") | 81 |

**Table 1. List of studies included and excluded after full-text analysis**

| **Lp.** | **Authors, Title, Journal** | **Full text status** | **Reason for exclusion** |
| --- | --- | --- | --- |
| **1.** | **Liang, Y., Deng, Z., Dai, X. et al. Micro-invasive interventions for managing non-cavitated proximal caries of different depths: a systematic review and meta-analysis. Clin Oral Invest 22, 2675–2684 (2018). https://doi.org/10.1007/s00784-018-2605-9** | **INCLUDED** | **–** |
| **2.** | **Bagheri E, Sarraf Shirazi A, Shekofteh K. Comparison of the Success Rate of Filled and Unfilled Resin-Based Fissure Sealants: A Systematic Review and Meta-Analysis. Front Dent. 2022 Feb 8;19:10. doi: 10.18502/fid.v19i10.8855. PMID: 35937149; PMCID: PMC9294718.** | **INCLUDED** | **–** |
| **3.** | **Kashbour W, Gupta P, Worthington HV, Boyers D. Pit and fissure sealants versus fluoride varnishes for preventing dental decay in the permanent teeth of children and adolescents. Cochrane Database of Systematic Reviews 2020, Issue 11. Art. No.: CD003067. DOI: 10.1002/14651858.CD003067.pub5.** | **INCLUDED** | **–** |
| **4.** | **Ahovuo-Saloranta A, Forss H, Walsh T, Nordblad A, Mäkelä M, Worthington HV. Pit and fissure sealants for preventing dental decay in permanent teeth. Cochrane Database of Systematic Reviews 2017, Issue 7. Art. No.: CD001830. DOI: 10.1002/14651858.CD001830.pub5** | **INCLUDED** | **–** |
| **5.** | **Alsabek L, Al-Hakeem A, Alagha MA, Comisi JC. Efficacy of hydrophilic resin-based sealant: A systematic review and meta-analysis. J Dent. 2021 Nov;114:103816. doi: 10.1016/j.jdent.2021.103816. Epub 2021 Sep 22. PMID: 34560227.** | **INCLUDED** | **–** |
| **6.** | **Li, F., Jiang, P., Yu, F. et al. Comparison between Fissure Sealant and Fluoride Varnish on Caries Prevention for First Permanent Molars: a Systematic Review and Meta-analysis. Sci Rep 10, 2578 (2020). https://doi.org/10.1038/s41598-020-59564-5** | **INCLUDED** | **–** |
| **7.** | **Kühnisch, J., Bedir, A., Lo, Y.-F., Kessler, A., Lang, T., Mansmann, U., … Hickel, R. (2020). Meta-analysis of the longevity of commonly used pit and fissure sealant materials. Dental Materials. doi:10.1016/j.dental.2020.02.001** | **INCLUDED** | **–** |
| **8.** | **Bagherian, A., & Shiraz, A. S. (2018). Flowable composite as fissure sealing material? A systematic review and meta-analysis. BDJ, 224(2), 92–97. doi:10.1038/sj.bdj.2018.40** | **INCLUDED** | **–** |
| **9.** | **Alirezaei, M., Bagherian, A., & Sarraf Shirazi, A. (2018). Glass ionomer cements as fissure sealing materials: yes or no? The Journal of the American Dental Association, 149(7), 640–649.e9. doi:10.1016/j.adaj.2018.02.001** | **INCLUDED** | **–** |
| **10.** | **Hou, J., Gu, Y., Zhu, L., Hu, Y., Sun, M., & Xue, H. (2015). Systemic review of the prevention of pit and fissure caries of permanent molars by resin sealants in children in China. Journal of Investigative and Clinical Dentistry, 8(1), e12183. doi:10.1111/jicd.12183** | **INCLUDED** | **–** |
| **11.** | **Yengopal, V., Mickenautsch, S. Resin-modified glass-ionomer cements versus resin-based materials as fissure sealants: a meta-analysis of clinical trials. Eur Arch Paediatr Dent 11, 18–25 (2010). https://doi.org/10.1007/BF03262705** | **INCLUDED** | **–** |
| **12.** | **Yengopal, V., Mickenautsch, S., Bezerra, A. C., & Leal, S. C. (2009). Caries-preventive effect of glass ionomer and resin-based fissure sealants on permanent teeth: a meta analysis. Journal of Oral Science, 51(3), 373–382. doi:10.2334/josnusd.51.373** | **INCLUDED** | **–** |
| **13.** | **Wright, J. T., Tampi, M. P., Graham, L., Estrich, C., Crall, J. J., Fontana, M., … Carrasco-Labra, A. (2016). Sealants for preventing and arresting pit-and-fissure occlusal caries in primary and permanent molars. The Journal of the American Dental Association, 147(8), 631–645.e18. doi:10.1016/j.adaj.2016.06.003** | **INCLUDED** | **–** |
| **14.** | **Mickenautsch S, Yengopal V. Caries-Preventive Effect of High-Viscosity Glass Ionomer and Resin-Based Fissure Sealants on Permanent Teeth: A Systematic Review of Clinical Trials. PLoS One. 2016 Jan 22;11(1):e0146512. doi: 10.1371/journal.pone.0146512. PMID: 26799812; PMCID: PMC4723148.** | **INCLUDED** | **–** |
| **15.** | **Muller-Bolla M, Lupi-Pégurier L, Tardieu C, Velly AM, Antomarchi C. Retention of resin-based pit and fissure sealants: A systematic review. Community Dent Oral Epidemiol. 2006 Oct;34(5):321-36. doi: 10.1111/j.1600-0528.2006.00319.x. PMID: 16948671.** | **INCLUDED** | **–** |
| 16. | De Amorim, R. G., Frencken, J. E., Raggio, D. P., Chen, X., Hu, X., & Leal, S. C. (2018). Survival percentages of atraumatic restorative treatment (ART) restorations and sealants in posterior teeth: an updated systematic review and meta-analysis. Clinical Oral Investigations. doi:10.1007/s00784-018-2625-5 | EXCLUDED | Treatment, not prevention (restoration, ART) |
| 17 | Jafarzadeh D, Rezapour R, Abbasi T, Sadegh Tabrizi J, Zeinolabedini M, Khalili A, Yousefi M. The Effectiveness of Fluoride Varnish and Fissure Sealant in Elementary School Children: A Systematic Review and Meta-Analysis. Iran J Public Health. 2022 Feb;51(2):266-277. doi: 10.18502/ijph.v51i2.8680. PMID: 35866130; PMCID: PMC9273487. | EXCLUDED | Not fully correct interpretation of test results |
| 18 | Dorri M, Dunne SM, Walsh T, Schwendicke F. Micro‐invasive interventions for managing proximal dental decay in primary and permanent teeth. Cochrane Database of Systematic Reviews 2015, Issue 11. Art. No.: CD010431. DOI: 10.1002/14651858.CD010431.pub2 | EXCLUDED | Deciduous teeth;  Treatment, not prevention (micro-invasive approaches) |
| 19 | Schwendicke F, Walsh T, Lamont T, Al-yaseen W, Bjørndal L, Clarkson JE, Fontana M, Gomez Rossi J, Göstemeyer G, Levey C, Müller A, Ricketts D, Robertson M, Santamaria RM, Innes NPT. Interventions for treating cavitated or dentine carious lesions. Cochrane Database of Systematic Reviews 2021, Issue 7. Art. No.: CD013039. DOI: 10.1002/14651858.CD013039.pub2. | EXCLUDED | Treatment, not prevention |
| 20 | Chen, Y., Chen, D. & Lin, H. Infiltration and sealing for managing non-cavitated proximal lesions: a systematic review and meta-analysis. BMC Oral Health 21, 13 (2021). https://doi.org/10.1186/s12903-020-01364-4 | EXCLUDED | Results combine infiltration with sealing for permanent teeth |
| 21. | Tamara Kerber Tedesco, Ana Flávia Bissoto Calvo, Ana Laura Pássaro, Mariana Pinheiro Araujo, Nathalia Miranda Ladewig, Samata Scarpini, Juan Sebastian Lara, Mariana Minatel Braga, Thais Gimenez & Daniela Prócida Raggio (2021): Nonrestorative treatment of initial caries lesion in primary teeth: a systematic review and network meta-analysis, Acta Odontologica Scandinavica, DOI: 10.1080/00016357.2021.1928748 | EXCLUDED | Treatment, not prevention |
| 22. | Ramamurthy P, Rath A, Sidhu P, Fernandes B, Nettem S, Fee PA, Zaror C, Walsh T. Sealants for preventing dental caries in primary teeth. Cochrane Database Syst Rev. 2022 Feb 11;2(2):CD012981. doi: 10.1002/14651858.CD012981.pub2. PMID: 35146744; PMCID: PMC8832104. | EXCLUDED | Deciduous teeth |
| 23. | Jha K.; Jain S.; Valluri B.P.; Jha S.; Dubey A.; Bhasker N. Retention of pit and fissure sealants with and without dentin bonding agents-a comparative systematic review. NeuroQuantology - Volume 20, Issue 15, pp. 3964-3970 | EXCLUDED | Lack of access |
| 24. | Rachna Mulani , Aditi Mathur. (2020). Retention of Various Pit and Fissure Sealants in Deciduous Teeth. A Systematic Review. Indian Journal of Public Health Research & Development, 11(7), 615–620. https://doi.org/10.37506/ijphrd.v11i7.10154 | EXCLUDED | Deciduous teeth |
| 25. | Taneja S, Singh A. Retention of flowable composite resins in comparison to pit and fissure sealants: a systematic review and meta-analysis. Gen Dent. 2020 Jul-Aug;68(4):50-55. PMID: 32597778. | EXCLUDED | Lack of access |
| 26. | Lam PPY, Sardana D, Ekambaram M, Lee GHM, Yiu CKY. Effectiveness of Pit and Fissure Sealants for Preventing and Arresting Occlusal Caries in Primary Molars: A Systematic Review and Meta-Analysis. J Evid Based Dent Pract. 2020 Jun;20(2):101404. doi: 10.1016/j.jebdp.2020.101404. Epub 2020 Jan 29. PMID: 32473795. | EXCLUDED | Deciduous teeth |
| 27. | Gugnani, N., & Gugnani, S. (2018). Sealants generally show equal performance regardless of tooth type and position. Evidence-Based Dentistry, 19(2), 40–41. doi:10.1038/sj.ebd.6401300 | EXCLUDED | Wrong publication type |
| 28. | Liu Y.; Xu Q.; Zhang F. Efficacy of light cured flowable composite resin and light cured pit and fissure sealant for prevention of dental caries in children: A meta-analysis. Chinese Journal of Evidence-Based Medicine - Volume 18, Issue 2, pp. 178-184 | EXCLUDED | Language (Chinese) |
| 29. | Schwendicke, F., Jäger, A. M., Paris, S., Hsu, L. Y., & Tu, Y. K. (2015). Treating Pit-and-Fissure Caries. Journal of Dental Research, 94(4), 522–533. doi:10.1177/0022034515571184 | EXCLUDED | Treatment, not prevention |
| 30. | Ammari, M. M., Soviero, V. M., da Silva Fidalgo, T. K., Lenzi, M., Ferreira, D. M. T. P., Mattos, C. T., … Maia, L. C. (2014). Is non-cavitated proximal lesion sealing an effective method for caries control in primary and permanent teeth? A systematic review and meta-analysis. Journal of Dentistry, 42(10), 1217–1227. doi:10.1016/j.jdent.2014.07.015 | EXCLUDED | Results for infiltration only |
| 31. | Ahovuo‐Saloranta A, Forss H, Walsh T, Hiiri A, Nordblad A, Mäkelä M, Worthington HV. Sealants for preventing dental decay in the permanent teeth. Cochrane Database of Systematic Reviews 2013, Issue 3. Art. No.: CD001830. DOI: 10.1002/14651858.CD001830.pub4. | EXCLUDED | A newer, updated version of the article was included in the analysis |
| 32. | Kühnisch J, Mansmann U, Heinrich-Weltzien R, Hickel R. Longevity of materials for pit and fissure sealing--results from a meta-analysis. Dent Mater. 2012 Mar;28(3):298-303. doi: 10.1016/j.dental.2011.11.002. Epub 2011 Dec 3. PMID: 22137936. | EXCLUDED | A newer, updated version of the article was included in the analysis |
| 33. | de Amorim, R.G., Leal, S.C. & Frencken, J.E. Survival of atraumatic restorative treatment (ART) sealants and restorations: a meta-analysis. Clin Oral Invest 16, 429–441 (2012). https://doi.org/10.1007/s00784-011-0513-3. | EXCLUDED | Treatment, not prevention (restoration, ART) |
| 34. | Azarpazhooh A, Main PA. Pit and fissure sealants in the prevention of dental caries in children and adolescents: a systematic review. J Can Dent Assoc. 2008 Mar;74(2):171-7. PMID: 18353204. | EXCLUDED | Results combine the deciduous teeth with permantent teeth |
| 35. | Hiiri A, Ahovuo-Saloranta A, Nordblad A, Mäkelä M. Pit and fissure sealants versus fluoride varnishes for preventing dental decay in children and adolescents. Cochrane Database Syst Rev. 2010 Mar 17;(3):CD003067. doi: 10.1002/14651858.CD003067.pub3. Update in: Cochrane Database Syst Rev. 2016;1:CD003067. PMID: 20238319. | EXCLUDED | A newer, updated version of the article was included in the analysis |
| 36. | Beiruti N, Frencken JE, van 't Hof MA, van Palenstein Helderman WH. Caries-preventive effect of resin-based and glass ionomer sealants over time: a systematic review. Community Dent Oral Epidemiol. 2006 Dec;34(6):403-9. doi: 10.1111/j.1600-0528.2006.00321.x. PMID: 17092268. | EXCLUDED | Insufficient description of the results |
| 37. | Ramesh H, Ashok R, Rajan M, Balaji L, Ganesh A. Retention of pit and fissure sealants versus flowable composites in permanent teeth: A systematic review. Heliyon. 2020 Sep 24;6(9):e04964. doi: 10.1016/j.heliyon.2020.e04964. PMID: 33005790; PMCID: PMC7519376. | EXCLUDED | Insufficient description of the results |
| 38. | Mejàre I, Lingström P, Petersson LG, Holm AK, Twetman S, Källestål C, Nordenram G, Lagerlöf F, Söder B, Norlund A, Axelsson S, Dahlgren H. Caries-preventive effect of fissure sealants: a systematic review. Acta Odontol Scand. 2003 Dec;61(6):321-30. doi: 10.1080/00016350310007581. PMID: 14960003. | EXCLUDED | Insufficient description of the results |
| 39. | Mickenautsch S, Yengopal V. Caries-preventive effect of glass ionomer and resin-based fissure sealants on permanent teeth: An update of systematic review evidence. BMC Res Notes. 2011 Jan 28;4:22. doi: 10.1186/1756-0500-4-22. PMID: 21276215; PMCID: PMC3041989. | EXCLUDED | A newer, updated version of the article was included in the analysis |
| 40. | Condò R, Cioffi A, Riccio A, Totino M, Condò SG, Cerroni L. Sealants in dentistry: a systematic review of the literature. Oral Implantol (Rome). 2014 Apr 4;6(3):67-74. PMID: 24772264; PMCID: PMC3982302. | EXCLUDED | Insufficient description of the results and included studies |
| 41. | de Assunção IV, da Costa Gde F, Borges BC. Systematic review of noninvasive treatments to arrest dentin non-cavitated caries lesions. World J Clin Cases. 2014 May 16;2(5):137-41. doi: 10.12998/wjcc.v2.i5.137. PMID: 24868513; PMCID: PMC4023307. | EXCLUDED | Treatment, not prevention |
| 42. | Griffin SO, Oong E, Kohn W, Vidakovic B, Gooch BF; CDC Dental Sealant Systematic Review Work Group; Bader J, Clarkson J, Fontana MR, Meyer DM, Rozier RG, Weintraub JA, Zero DT. The effectiveness of sealants in managing caries lesions. J Dent Res. 2008 Feb;87(2):169-74. doi: 10.1177/154405910808700211. PMID: 18218845. | EXCLUDED | Treatment, not prevention |

**Details on the methodology of the included systematic reviews**

- Bagheri 2022 – a systematic review of 19 RCTs (Randomised Controlled Trial) with meta-analysis (26 comparison groups) comparing the efficacy of filled and unfilled fissure sealants in terms of retention and prevention of caries development;
- Alsabek 2021 – a systematic review of 10 RCTs with meta-analysis evaluating the efficacy of hydrophilic resin-based fissure sealants (hRBS) in preventing and stopping further development of caries in fissures and pits of permanent teeth and comparing the retention rate of hRBS with alternative methods of caries prevention;
- Kashbour 2020 – a systematic review of 11 RCTs with meta-analysis comparing the relative efficacy of resin-based fissure sealants with fluoride varnishes in the prevention of caries on occlusal surfaces of permanent teeth in children and adolescents;
- Kühnisch 2020 – a systematic review of 51 experimental studies (clinical trials – CT) with meta-analysis comparing retention rates when using prime sealants and conventional fissure sealants (auto-polymerising, light-polymerising and fluoride-releasing sealants);
- Li 2020 – a systematic review of 8 RCTs with meta-analysis comparing the efficacy of fissure sealants with fluoride-releasing sealants in the prevention of caries in permanent first molars;
- Alirezaei 2018 – a systematic review of 31 RCTs with meta-analysis, evaluating the efficacy of glass-ionomer cements (GICs) and resin-based sealants (RBS) for caries prevention and retention maintenance of these materials;
- Bagherian 2018 – a systematic review of 11 RCTs with meta-analysis comparing the retention rate of flowable composites used as fissure sealants compared with conventional resin-based fissure sealants;
- Liang 2018 – a systematic review of 8 RCTs with meta-analysis, evaluating the effectiveness of micro-invasive interventions for caries prevention on proximal surfaces of non-cavitated proximal caries teeth;
- Ahovuo-Saloranta 2017 – a systematic review of 38 RCTs with meta-analysis comparing the effectiveness of different types of fissure sealants in the prevention of caries on the occlusal surfaces of permanent teeth in children and adolescents;
- Mickenautsch 2016 – a systematic review of 7 CTs (11 datasets) with meta-analysis comparing the effectiveness of high-viscosity glass ionomer with resin-based fissure sealants in the prevention of caries in fissures and cavities on the occlusal surfaces of permanent teeth;
- Wright 2016 – a systematic review of 23 RCTs with meta-analysis (American Dental Association and the American Academy of Pediatric Dentistry – ADA/AAPD) summarising the available clinical data on the effect of fissure sealants on the prevention and treatment of caries on the occlusal surface of permanent molars compared to a control group with no sealant or other preventive methods;
- Hou 2015 – a systematic review of 20 RCTs with meta-analysis, determining the efficacy of pits and fissure sealing for caries prevention of permanent molars in children in China;
- Yengopal 2010 – a systematic review of 6 RCTs (19 datasets) with meta-analysis, determining the efficacy in caries prevention when resin-modified glass-ionomer cement (RM-GIC) is used in fissure sealing compared to resin-based fissure sealants;
- Yengopal 2009 – a systematic review of 8 RCTs and 3 systematic reviews with meta-analysis, determining the caries prevention efficacy of resin-modified glass-ionomer cements (GICs) compared to RBS when used in fissure sealing;
- Muller-Bolla 2006 – a systematic review of 16 RCTs with meta-analysis, assessing the total retention rate of resin-based fissure sealers (RBS) depending on the type of material used.

**AMSTAR2**

The systematic reviews included in the analysis received the following ratings:

- high – Kashbour 2020, Ahovuo-Saloranta 2017, Mickenautsch 2016;
- low – Alsabek 2021;
- critically low – Begheri 2022, Kuhnish 2020, Li 2020, Alirezaei 2018, Begherian 2018, Liang 2018, Wright 2016, Hou 2015, Yengopal 2010, Yengopal 2009, Muller-Bolla 2006.

| **Publication** | **Item 2** | **Item 4** | **Item 7** | **Item 9** | **Item 11** | **Item 13** | **Item 15** | **Overall rating** |
| --- | --- | --- | --- | --- | --- | --- | --- | --- |
| Begheri 2022 (MA) | No | Yes | No | Yes | Yes | Yes | Yes | **Critically low** |
| Item 2: the authors did not demonstrate that they had created a written protocol or guide before proceeding with the review.  Item 7: the authors did not provide a list of excluded studies. | | | | | | | | |
| Alsabek 2021 (MA) | Partial Yes | Partial Yes | No | Yes | Yes | Yes | Yes | **Low** |
| Item 2: the authors did not provide a plan for investigating causes of heterogeneity in protocol.  Item 4: The authors did not indicate whether they had searched gray literature.  Item 7: the authors did not provide a list of excluded studies. | | | | | | | | |
| Kashbour 2020 (MA) | Yes | Yes | Yes | Yes | Yes | Yes | Yes | **High** |
| No objections. | | | | | | | | |
| Kuhnish 2020 (MA) | No | Partial Yes | No | No | Yes | No | Yes | **Critically low** |
| Item 2: The authors failed to demonstrate that they had created a written protocol or guide before proceeding with the review.  Item 4: the authors did not indicate whether they had searched the reference list/bibliographies of included studies and did not indicate whether they had searched gray literature.  Item 7: the authors did not provide a list of excluded studies.  Item 9: A satisfactory technique for assessing the risk of bias in individual studies has not been presented. The authors of the study did not use a properly developed rating instrument.  Item 13: the authors did not take into account the risk of bias in individual studies when interpreting/discussing the results of the review. | | | | | | | | |
| Li 2020 (MA) | No | Partial Yes | Yes | Yes | Yes | Yes | No | **Critically low** |
| Item 2: The authors failed to demonstrate that they had created a written protocol or guide before proceeding with the review.  Item 4: the authors did not indicate whether they had searched the reference list/bibliographies of included studies and did not indicate whether they had searched gray literature.  Item 15: publication bias was not addressed in the review. | | | | | | | | |
| Alirezaei 2018 (MA) | No | Partial Yes | No | Yes | Yes | Yes | Yes | **Critically low** |
| Item 2: The authors failed to demonstrate that they had created a written protocol or guide before proceeding with the review.  Item 4: The authors did not indicate whether they had searched gray literature.  Item 7: the authors did not provide a list of excluded studies. | | | | | | | | |
| Begherian 2018 (MA) | No | Partial Yes | No | Yes | Yes | Yes | No | **Critically low** |
| Item 2: The authors failed to demonstrate that they had created a written protocol or guide before proceeding with the review.  Item 4: the authors did not indicate whether they had searched the reference list/bibliographies of included studies and gray literature.  Item 7: the authors did not provide a list of excluded studies.  Item 15: publication bias was not sufficiently addressed. It was only indicated that "based on the recommendations from the Cochrane Handbook for Systematic Reviews of Intervention, if we found more than ten studies to be included in the meta-analysis, then we will explore publication bias by using funnel plots". Although the study did not include more than 10 studies, publication bias was not addressed at any other point in the study. | | | | | | | | |
| Liang 2018 (MA) | No | Partial Yes | No | Yes | Yes | Yes | Yes | **Critically low** |
| Item 2: The authors failed to demonstrate that they had created a written protocol or guide before proceeding with the review.  Item 4: the authors did not indicate whether they had searched the reference list/bibliographies of included studies and did not indicate whether they had searched gray literature.  Item 7: the authors did not provide a list of excluded studies. | | | | | | | | |
| Ahovuo-Saloranta 2017 (MA) | Yes | Yes | Yes | Yes | Yes | Yes | Yes | **High** |
| No objections. | | | | | | | | |
| Mickenautsch 2016 (MA) | Yes | Yes | Yes | Yes | Yes | Yes | Yes | **High** |
| No objections. | | | | | | | | |
| Wright 2016 (MA) | No | Partial Yes | No | Yes | Yes | Yes | Yes | **Critically low** |
| Item 2: The authors failed to demonstrate that they had created a written protocol or guide before proceeding with the review.  Item 4: The authors did not indicate whether they had searched gray literature.  Item 7: the authors did not provide a list of excluded studies. | | | | | | | | |
| Hou 2015 (MA) | No | Partial Yes | No | Yes | Yes | Yes | Yes | **Critically low** |
| Item 2: The authors failed to demonstrate that they had created a written protocol or guide before proceeding with the review  Item 4: the authors did not indicate whether they had searched the reference list/bibliographies of included studies and did not indicate whether they had searched gray literature.  Item 7: the authors did not provide a list of excluded studies. | | | | | | | | |
| Yengopal 2010 (MA) | No | Partial Yes | Yes | Partial Yes | Yes | Yes | No | **Critically low** |
| Item 2: The authors failed to demonstrate that they had created a written protocol or guide before proceeding with the review.  Item 4: the authors did not indicate whether they had searched the reference list/bibliographies of included studies and did not indicate whether they had searched gray literature.  Item 9: the authors did not carry out a risk of bias analysis from selection of the reported result from among multiple measurements or analyzes of a specified outcome.  Item 15: publication bias was not addressed in the review. | | | | | | | | |
| Yengopal 2009 (MA) | No | Partial Yes | Yes | Partial Yes | Yes | Yes | No | **Critically low** |
| Item 2: The authors failed to demonstrate that they had created a written protocol or guide before proceeding with the review.  Item 4: the authors did not indicate whether they had searched the reference list/bibliographies of included studies and did not indicate whether they had searched gray literature.  Item 9: the authors did not carry out a risk of bias analysis from selection of the reported result from among multiple measurements or analyzes of a specified outcome.  Item 15: publication bias was not addressed in the review. | | | | | | | | |
| Muller-Bolla 2006 (MA) | No | Yes | Yes | Yes | Yes | Yes | No | **Critically low** |
| Item 2: The authors failed to demonstrate that they had created a written protocol or guide before proceeding with the review.  Item 15: Authors did not adequately investigate publication bias. | | | | | | | | |

MA – meta-analysis

*Critical domains: item 2 – protocol registered before commencement of the review; item 4 – adequacy of the literaturę search; item 7 – justyfication for excluding individual studies; item 9 – risk of bias from individual studies being included in the review; item 11 – appropriateness of meta-analytical methods; item 13 – consideration of risk of bias when interpreting the results of the review; item 15 – assessment of presence and likely impact of publication bias.*
